# A WASN-Based Suburban Dataset for Anomalous Noise Event Detection on Dynamic Road-Traffic Noise Mapping

**DOI:** 10.3390/s19112480

**Published:** 2019-05-30

**Authors:** Rosa Ma Alsina-Pagès, Ferran Orga, Francesc Alías, Joan Claudi Socoró

**Affiliations:** GTM—Grup de recerca en Tecnologies Mèdia, La Salle—Universitat Ramon Llull., C/Quatre Camins, 30, 08022 Barcelona, Spain; ferran.orga@salle.url.edu (F.O.); francesc.alias@salle.url.edu (F.A.); joanclaudi.socoro@salle.url.edu (J.C.S.)

**Keywords:** road-traffic noise, anomalous noise event, acoustic dataset, noise monitoring, smartcity, WASN, SNR, impact, *L_Aeq_*, urban sound, noise maps

## Abstract

Traffic noise is presently considered one of the main pollutants in urban and suburban areas. Several recent technological advances have allowed a step forward in the dynamic computation of road-traffic noise levels by means of a Wireless Acoustic Sensor Network (WASN) through the collection of measurements in real-operation environments. In the framework of the LIFE DYNAMAP project, two WASNs have been deployed in two pilot areas: one in the city of Milan, as an urban environment, and another around the city of Rome in a suburban location. For a correct evaluation of the noise level generated by road infrastructures, all Anomalous Noise Events (ANE) unrelated to regular road-traffic noise (e.g., sirens, horns, speech, etc.) should be removed before updating corresponding noise maps. This work presents the production and analysis of a real-operation environmental audio database collected through the 19-node WASN of a suburban area. A total of 156 h and 20 min of labeled audio data has been obtained differentiating among road-traffic noise and ANEs (classified in 16 subcategories). After delimiting their boundaries manually, the acoustic salience of the ANE samples is automatically computed as a contextual Signal-to-Noise Ratio (SNR) together with its impact on the A-weighted equivalent level ($\Delta L_{Aeq}$). The analysis of the real-operation WASN-based environmental database is evaluated with these metrics, and we conclude that the 19 locations of the network present substantial differences in the occurrences of the subcategories of ANE, with a clear predominance of the noise of sirens, trains, and thunder.

## 1. Introduction

Presently, cities are growing in both size and population, and the consequent increase in vehicles is making traffic noise problem more present, with a clear effect on the quality of life of their citizens [1]. Noise is one of the main environmental health concerns [2,3], and its impact on social and economic aspects has been proved [4]. To face this issue, European authorities have driven the European Noise Directive (END) [5], focused on the creation of noise-level maps to inform citizens of their exposure to noise, and aided the authorities to take appropriate action to minimize its impact.

Noise maps have been historically generated by means of costly expert measurements using certified sound-level meters, with a basis of short-term periods aimed at being sufficiently representative. This approach is presently overcome by the technological advances of the Internet of Things in the framework of smart cities, which has allowed the emergence of Wireless Acoustic Sensor Networks (WASN) [6,7]. In the literature, several different WASNs have been designed for urban sound monitoring, some of them focused on security and surveillance and others on city noise management, involving noise mapping, the development of action plans, and public awareness campaigns. For example, the SENSEable project [8] deployed a WASN to collect information from the acoustic environment by means of a set of low-cost acoustic sensors with the goal of analyzing that data together with public health information. Other similar projects are the IDEA project in Belgium [9], or the RUMEUR network in France [10] with special focus on aircraft noise, or even the Barcelona noise-monitoring network [11], whose data is integrated in the Sentilo city management platform [12]. Recently, the SONYC project has deployed 56 low-cost acoustic sensors across New York City to monitor urban noise and perform a multi-label classification of urban sound sources in real time [4], but not on site. Finally, the LIFE DYNAMAP project [13] aims to monitor the noise level generated by road infrastructures by means of two WASNs installed in two pilot areas, one within an urban environment in Milan (District 9), and another in a suburban area surrounding Rome (A90 highway). To monitor Road-Traffic Noise (RTN) levels reliably, all Anomalous Noise Events (ANE) unrelated to regular RTN (e.g., sirens, horns, speech, etc.) should be removed before updating the corresponding noise maps [13].

The deployment of these projects has shown that the WASN paradigm entails several challenges, from technical issues [14,15], to other aspects related to the WASN-based application, such as the automation of data collection and the subsequent signal processing [16,17,18], especially if the system intends detect acoustic events in real operation and locally in each sensor. Acoustic event detection and classification belongs to the Computational Auditory Scene Analysis (CASA) paradigm [19], and it is usually based on the segmentation of the input acoustic data into slices that represent a single occurrence of the target class, and focus on individual simultaneous events [20]. To do so, Acoustic Event Detection (AED) algorithms are typically trained with databases designed ad hoc in each of the problems to be solved, hence typically considering a finite set of predefined acoustic classes [21,22].

Therefore, the development of AED-based applications entails representative audio databases with all kinds of sounds of interest, as in the one obtained in the SONYC project [4], with data from 56 sensors deployed in different neighborhoods of New York, which considers 10 different kinds of common urban sound sources labeled in an urban soundscape. As a first attempt to create an acoustic dataset to model the acoustic environments of urban and suburban pilot areas in the framework of the DYNAMAP project, an expert-based recording campaign was conducted before the two WASNs were deployed [23]. The analyses showed the highly local, unpredictable, and diverse nature of ANEs in real acoustic environments can be far different to previous models obtained by means of synthetically generated datasets [23]. After labeling the gathered acoustic data, the dataset was used to train the AED-based algorithm designed to detect ANEs, known as Anomalous Noise Event Detector (ANED) [18]. Although that preliminary dataset collected a representative number of acoustic events of interest from both acoustic environments, it missed several key aspects, such as different RTN patterns observed during day–night, weekday–weekend and the effect of diverse weather conditions [24]. This work describes the generation of the acoustic dataset to model the Rome’s acoustic environment in real-operation conditions, after deploying the 19-node WASN in its final location. The paper describes the conducted recording campaign and the subsequent labeling of ANEs in 16 different subcategories (without considering combined sounds in a sample), as well as the analysis of their occurrences, duration, Signal-to-Noise Ratio (SNR), and impact on the A-weighted equivalent noise level ($L_{Aeq}$ ) computation.

The remainder of this paper is the following. Section 2 details the most relevant previous attempts to generate environmental audio databases. Section 3 describes the generation and labeling of the real-operation conditions environmental audio database in the suburban scenario. Section 4 analyses the ANE of the dataset in terms of occurrences, duration, SNR, and impact on the $L_{Aeq}$. Finally, Section 5 discusses in detail the results obtained in the analysis and the future applications of the designed dataset.

## 2. Related Work

In the literature, several audio databases related to machine hearing (or machine listening) have been unveiled for benchmarking purposes under the umbrella of the so-called CASA, and mainly oriented to evaluate the performance of acoustic scene classification and AED. This section reviews the literature about environmental acoustic databases and the datasets designed for challenges (e.g., DCASE), and describes their characteristics and limitations.

### 2.1. Environmental Acoustic Databases

The environmental acoustic databases employed by the machine-hearing research community have been generally created from live recordings directly and/or synthetically generated by artificially mixing sound events with certain acoustic environments (i.e., background noise). The latter allows control of the SNR of the mixture, and dealing with data scarcity of specific audio events in real-life contexts—which is one of the key problems when trying to gather representative data from live environments [25]—while the former entails a huge effort for data collection and subsequent manual annotation to generate the labeled database or ground truth [23,26,27].

Regarding real-life environmental acoustic databases, in [28] a 1133-min audio database including 10 different acoustic environments, both indoor and outdoor was introduced. On the other hand, the MIVIA audio events dataset was designed for surveillance applications focused on the identification of glass breaking, gun shots, and screams (https://mivia.unisa.it/datasets/audio-analysis/mivia-audio-events/) [21]. The training dataset is about 20 h, while the test set is about 9 h. Moreover, the same research laboratory developed a smaller dataset of about 1 h duration also for surveillance purposes focused on road audio events, which contains sound events from tire skidding and car crashes (https://mivia.unisa.it/datasets/audio-analysis/mivia-road-audio-events-data-set/) [29].

In [23], a real-life acoustic database collected from the urban and suburban environments of the pilot areas of the Life DYNAMAP project [13] is described. The database composed of 9 h and 8 min was obtained through an in situ recording campaign. This acoustic database was developed for discriminating road-traffic noise from ANE through the ANED [18]. The ANEs, which only represented 7.5% of the annotated data, were subsequently classified in 19 different subcategories after manual inspection, showing SNR levels with respect to background noise that ranged from −10 dBm to +15 dBm and showing a high heterogeneity of intermediate SNR levels. When comparing both environments, it can be observed that the number of ANEs in the urban area is approximately four times higher than in the suburban area, also including events with larger acoustic salience. Nevertheless, it is worth mentioning that the recordings in the urban area were conducted at the street level of the preselected locations [30] within District 9 of Milan, while the recordings in the suburban area were conducted on the A90 ring-road portals surrounding Rome (see [31] for further details).

One of the main sources employed to build acoustic databases is the well-known Freesound online repository (https://www.freesound.org). For instance, *freefield1010* is a database composed of 7690 audio clips tagged as “field recording” in the metadata of the original recordings uploaded in this online repository, totaling over 21 h of audio [32]. In [33], 60 h of real field recordings uploaded in Freesound from urban environments were used to build the UrbanSound database. The database is composed of 27 h, which includes 18.5 h of verified and annotated sound event occurrences classified in 10 sound categories (i.e., air conditioner, car horn, children playing, dog bark, drilling, engine idling, gun shot, jackhammer, siren, and street music). Moreover, the authors also provide an 8.75 h subset—denoted as UrbanSound8k—designed to train sound classification algorithms and obtained after arbitrarily fixing the number of items to 1000 slices per class. In [34], a mixture of sound sources from Freesound mixed with real-life traffic noise was considered to train and evaluate an AED algorithm (considering two SNRs levels: +6 dB and +12 dB). Finally, “ESC: Dataset for Environmental Sound Classification” [35] is composed of three subsets: *(i)* ESC-50, a strictly balanced 50 classes of various environmental sounds obtained through manual annotation, *(ii)* ESC-10, as a reduced 10 classes subset of the former as a proof-of-concept dataset, and *(iii)* EC-US, which contains 250,000 recordings directly extracted from the “field recording”-tagged category of Freesound. Nevertheless, due to the uncontrolled origin of the sound sources uploaded to Freesound and similar online repositories (see [23] for further examples), involving a wide variation in the recording conditions and quality, the derived environmental acoustic databases may become unsuitable for reliable CASA- and AED-based systems evaluation purposes [27,32].

In [27], the described acoustic dataset covers both indoor and outdoor environments, including real-life recordings of predefined acoustic event sequences and individual acoustic events synthetically mixed with background recordings by considering specific SNRs levels, such as −6 dB, 0 dB, and +6 dB. In [25], the authors developed a mixed acoustic database composed of acoustic data from real-life recordings, which was subsequently extended with synthetic mixtures of extra events of interest to increase database diversity. In [21], an acoustic database for surveillance purposes that includes sound events such as screams, glass breaking, and gunshots was also artificially generated from indoor and outdoor environments considering different SNR levels (from +5 to +30 dB). Moreover, a small environmental acoustic database containing 20 scenes mixing background noise with car, bird, and car horn samples synthesized with SimScene software [36] was described in [37]. Following a similar approach, in [38], the TUT Sound Events Synthetic 2016 (TUT-SED-2016 for short) was introduced. The 566 min dataset is composed of synthetic mixtures created by mixing isolated sound events from 16 sound event classes from the original TUT database (The reader is referred to http://www.cs.tut.fi/sgn/arg/taslp2017-crnn-sed/tut-sed-synthetic-2016 for a detailed explanation).

Finally, it is worth mentioning the recent development of an open-source library for the synthesis of soundscapes named Scaper, mainly focused on SED-related applications [39]. This library provides an audio sequencer to generate synthetic soundscapes following a probabilistic approach including isolated sound events. The proposal allows control of the characteristics of the sound mixtures, such as the number of events, and their type, timing, duration, and SNR level with respect to the background noise. The authors validate their proposal through the development of the URBAN-SED database from UrbanSound8k as an example of the result of this kind of data augmentation. The synthetic generation of acoustic databases is a potential solution to address data scarcity when training Deep Neural Networks (DNN) for CASA-related problems (e.g., see [38,40]). Nevertheless, although the artificial generation of sound mixtures allows the creation of controlled training and evaluation environments, it has been also stated that these artificially generated databases could not represent the variability encountered in real-life environments accurately enough [18,26].

### 2.2. Challenge-Oriented Acoustic Datasets

Over the last decade, the CASA research community has provided publicly available datasets and standard metrics to evaluate the development of their investigations. One of the seminal international efforts that emerged to evaluate systems developed to model the perception of people, their activities, and interactions was the CLassification of Events, Activities and Relationships (CLEAR) competition, which included in its 2006 and 2007 editions specific data for acoustic event detection and classification mainly collected from indoor environments—specifically, from meeting rooms (see e.g., [41,42,43]). Although other attempts emerged to provide evaluation material for CASA-focused systems, such as DARESounds.org initiative [44] or TRECVID Multimedia Event Detection competition (focused on audiovisual and multi-modal event detection (https://www.nist.gov/itl/iad/mig/multimedia-event-detection)), neither them nor CLEAR led to the establishment of a reference evaluation challenge for the CASA research community [20].

Later, the IEEE AASP supported a new competition named Detection and Classification of Acoustic Scenes and Events (DCASE), which started in 2013 at WASPAA conference (http://www.waspaa.com/waspaa13/d-case-challenge/index.html). The database that was provided for that challenge contained both live and synthetic recordings [45]. The results of that competition can be found in [27]. Since 2016, DCASE has become an annual competition including different challenges covering acoustic scene classification, sound event detection in synthetic and in real-life audio, domestic audio tagging, or the detection of bird or rare sound events, to name a few (see [22,46,47] for further details) (http://dcase.community/events), mainly thanks to the contribution of several researchers who have made available different datasets for public evaluation.

Among them, it is worth mentioning the creation of the “TUT Database for Acoustic Scene Classification and Sound Event Detection” from real-life recordings [26]. This database allows the evaluation of automatic event or acoustic scene detection systems within 15 different real-life acoustic environments, such as lakeside beach, bus, cafe/restaurant, car, city center, forest path, grocery store, home, library, metro station, office, urban park, residential area, train, and tram. It is worth mentioning that the sound events subset, which covers both indoor and outdoor environments, it is mainly focused on surveillance and human activity monitoring. Finally, it is worth noting that in the recent announcement of the DCASE2019 competition, an Urban Sound Tagging (UST) challenge has been presented. The goal of this task is to predict whether each of 23 sources of noise pollution is present or absent in a 10-s scene. In this challenge, the audio in the dataset has been acquired with the acoustic sensor network of the SONYC project: SOunds of New York City [4], and it provides a simplified taxonomy of the sounds of the city in two levels, 8 coarse categories, and 23 fine labels. The challenge dataset includes 2351 recordings in the training split and 443 in the validation split, making a total of 2794 10-s audios. The full taxonomy and details of the SONYC project dataset can be found in [33].

## 3. Design of an Environmental Database

In this section, the real-operation environmental audio database recorded and built in for the suburban area of the DYNAMAP project is detailed. First, the conducted recording campaign is described. Second, the subsequent generation of the audio database is detailed, which includes the labeling and the computation of the acoustic salience of the anomalous noise events (in terms of SNR) and their impact on the $L_{Aeq}$. These are computed in this study and used jointly with duration, number of occurrences and other database descriptors to analyze in detailed nature of the ANEs of the suburban area.

### 3.1. Description of the WASN-based Suburban Recording Campaign

The main goal of any recording campaign is to collect representative samples of the acoustic environment under study through the WASN in real operation. Taking advantage of the experience gained from the preliminary recording and analysis of that acoustic environment [23], a second recording campaign was designed. The main reason for it was three-fold: *(i)* all the nodes of the WASN had been already deployed in their definitive operative location, which increased the number of recording points and changed slightly the sensor position in the portals, *(ii)* the sampling completeness, because the previous recording campaign did not include nighttime, weekend data, or different meteorological conditions, and *(iii)* the total amount of time recorded was quite short (4 h and 44 min), including only 12.2% of ANEs, which led us to the conclusion that ANEs were misrepresented in the dataset, after discarding the augmentation of the dataset by means of synthetic samples according to [23].

**Figure 1:**
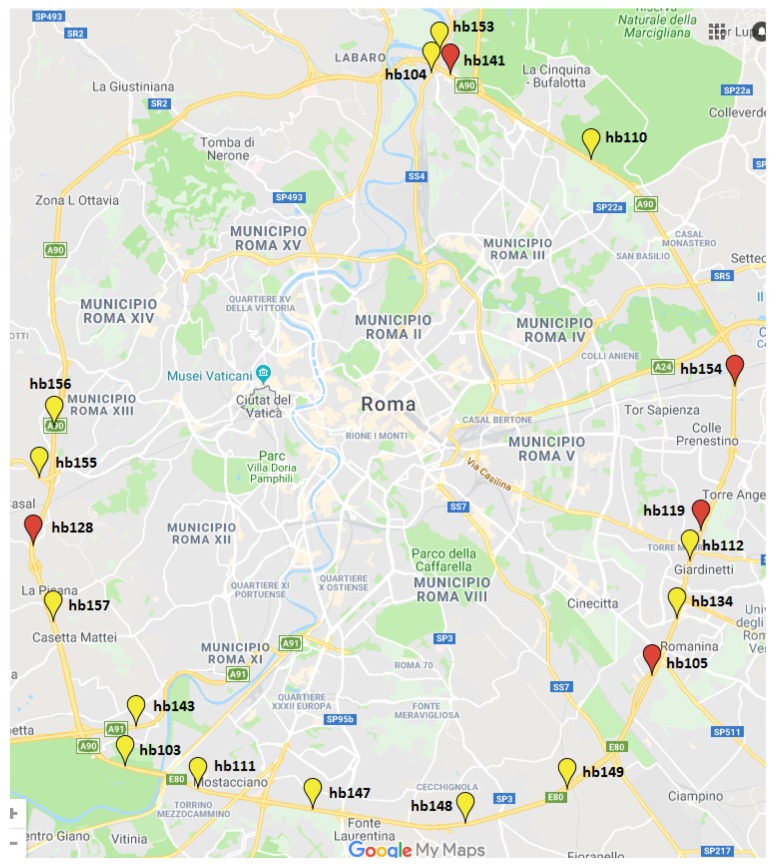
Map with sensors’ location information within the WASN of DYNAMAP project in the suburban area of Rome, those locations also sensed during the initial recording campaign described in [23] shown in red.

The WASN deployed in the suburban area of DYNAMAP project is located on the A90 highway surrounding Rome, and comprises 24 acoustic nodes, 5 of which are low-capacity sensors without enough computational resources to run the ANED algorithm. The locations of the 19 high-capacity sensors of the WASN in the Rome’s suburban pilot area are shown in Figure 1. The set of basic specifications [48] that are defined to satisfy DYNAMAP requirements for each monitoring station are the following: *(i)* 40–100 dB(A) broadband linearity range, *(ii)* 35–115 dB working range with acceptable Total Harmonic Distortion (THD), and *(iii)* narrowband floor noise level. The project also requires the possibility of audio recording, as well as Virtual Private Network (VPN) connection and GPRS/3G/WiFi connection. The precision of the sensors is a key issue for the system reliability [49]. During the developing stage, all the elements that could increase the uncertainty of the measurement were taken into account following the requirements of IEC 61672 [50]. Several tests have been conducted with both the hardware and the software, using a climate chamber with different operation temperatures. Electromagnetic Compatibility tests were also conducted as well as atmospheric agent simulations over the designed equipment [51].

**Figure 2:**
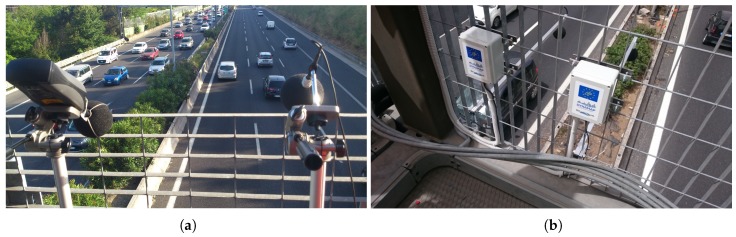
Location of the sensor used in the recording campaign in the portals. (**a**) Recording campaign deployment in [23]; (**b**) WASN deployment in this work (picture property of ANAS S.p.A.).

Some of the recording locations are the same as the ones used in the previous recording campaign [23] (see Figure 2). However, although conducted in the same portal both the sensor and the exact location measurement differ. The microphone location is slightly different with respect to the entire structure of the portal (see Figure 2a,b).

**Figure 3:**
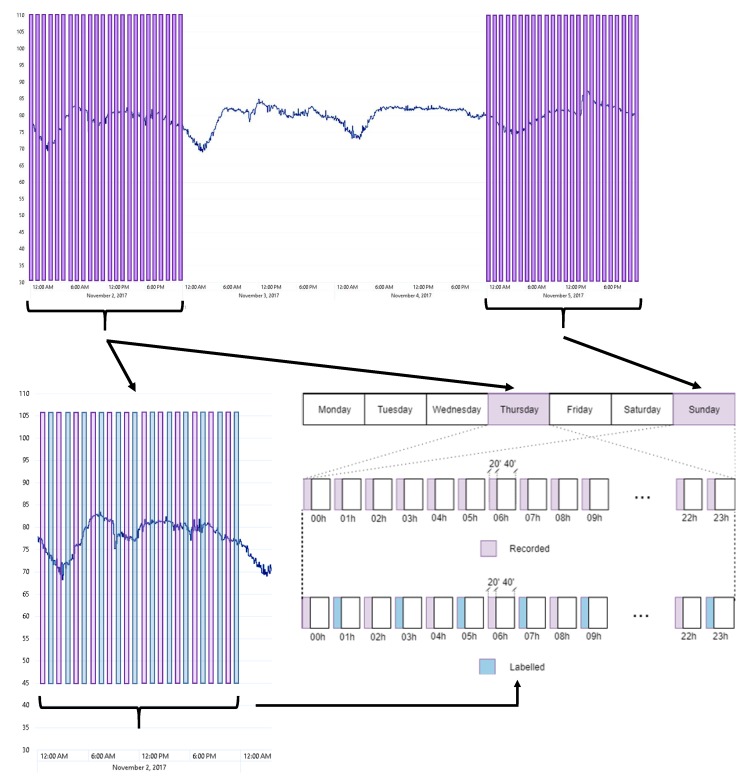
Daily curve of $L_{Aeq}$ for sensor hb147 for the 2nd and the 5th of November 2017, and the recordings conducted. Diagram of the recording days and duration for each sensor, and scheme of the labeled files to build the dataset.

Achieving a complete and exhaustive dataset is a challenging task since the amount of available resources is limited, e.g., processing and storage capabilities, data collection using 3G modems, availability of all nodes in fully operative conditions, etc. In Figure 3, it can be observed that the $L_{Aeq}$ presents a diurnal variation that suggests sampling the recording differentiating day and night to obtain data from several patterns of traffic noise, and so of ANEs.

To this aim, one-day real-operation recordings were planned through all nodes of the suburban WASN considering different days of variating traffic conditions, and assuming a trade-off between completeness and available computation and data communication resources, which were limited by the resources available in each of the nodes of the WASN (storage capacity, throughput of data, etc.). The following data sampling approach was proposed: Thursday and Sunday were selected as representative weekday and weekend days, respectively, being the 2nd and the 5th of November 2017, specifically. From each sensor, 20 min have been recorded per hour which was limited by the storage capacity and communication resources of each of the nodes. Figure 3 shows the recordings over the values of $L_{Aeq}$, while Figure 3 shows a schematic diagram with the recording process during the selected weekday and weekend day. As a result, 16 h of acoustic data were collected from each sensor to cover the diversity of the acoustic environment in a workday and a weekend day in this suburban environment.

It is worth mentioning that the high-capacity sensors used to conduct the recording campaign using the WASN in real operation were low-cost acoustic sensors designed ad hoc for the DYNAMAP project [51].

### 3.2. Data Labeling

After recording representative acoustic data for building the suburban environmental database, a labeling process was conducted. The manual annotation of ANEs becomes particularly complex when dealing with real-operation data from raw recordings. Thus, this process must be conducted by experts, since it is very important to precisely determine the occurrence and boundaries of each event, e.g., indicating the start and end points in the mixed audio [23].

The labeling process was not exhaustive because of the excessive burden that such a task would represent if considering all recorded data, meaning that only 50% of gathered audio signals were finally labeled. This represented a total of 156 h and 20 min of labeled audio data. From the labeling process 94.8% was labeled as RTN, 1.8% as ANE and the remaining 3.4% was categorized as *others* when the audio passage was difficult to categorize in one or other class due to the complexity of the audio scene. These last passages were not included in the subsequent analyses presented throughout this paper, but they have been left for future analyses that will focus, e.g., on the impact they can have for ANED assessment.

**Figure 4:**
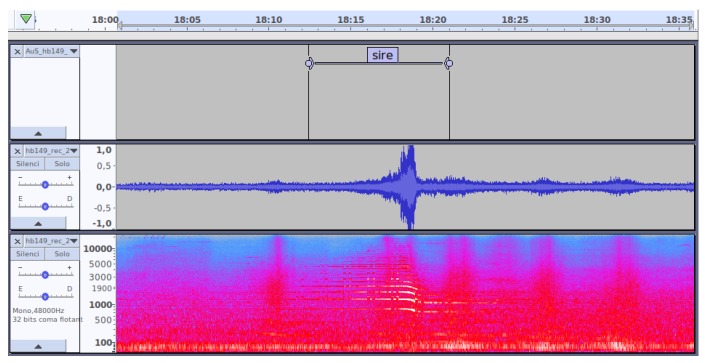
Example of the labeling of a siren using Audacity, with the label on top, the signal in the middle and the spectrum on the bottom.

In Figure 4, an example of the labeling process using Audacity software is shown for illustrative purposes. The example of ANE is a siren, which is a long event, recorded in sensor hb149 the 2nd of November 2017.

From the labeling of all the collected data through the 19 nodes of the Rome’s WASN, the following list of ANEs were observed:

- *airp*: airplanes.
- *alrm*: sounds of cars and houses alarm systems.
- *bike*: noise of bikes.
- *bird*: birdsong.
- *brak*: noise of brake or cars’ trimming belt.
- *busd*: opening bus or tramway, door noise, or noise of pressurized air.
- *door*: noise of house or vehicle doors, or other object blows.
- *horn*: horn vehicles noise.
- *inte*: interfering signal from ad industry or human machine.
- *musi*: music in car or in the street.
- *rain*: sound produced by heavy rain.
- *sire*: sirens of ambulances, police, fire trucks, etc.
- *stru*: noise of portals structure derived from its vibration, typically caused by the passing-by of very large trucks.
- *thun*: thunderstorm.
- *tran*: stop, start, and pass-by of trains.
- *trck*: noise when trucks or vehicles with heavy load passed over a bump.

Another label has been used for the annotation process, the *cmplx* label that indicates that the piece of raw acoustic audio was the result of more than one subcategory of ANE or that the sound was not identifiable by the labeler. This is not considered to be a subcategory of ANE because it cannot be assigned to a single noise source.

### 3.3. Characterization of the ANEs

In previous works, two parameters were considered by the authors to figure out the effects of the ANEs on noise-map generation [52]. The first of the parameters is based on the classical SNR calculation, consisting of the ratio of power of the ANE in relation to the power of the surrounding RTN. The second metric determines the impact of the ANE on the equivalent noise level used to build the noise map. The calculation of the two parameters is described below.

#### 3.3.1. SNR Calculation

As aforementioned, the SNR is calculated as the classical signal-to-noise ratio used in signal processing, considering that the ANE corresponds to the signal and the RTN is the noise. The acoustic power of the ANE and the RTN are calculated as follows:

(1) $$P_{x}=\sum_{n=1}^{N}\left(\frac{x{\left[n\right]}^{2}}{N}\right)$$

where x[n] is the recorded audio with *N* samples that belongs to either the ANE of the RTN.

After the power calculations of the ANE and the surrounding RTN, the SNR is calculated as:

(2) $$SNR=10\;log_{10}\left(\frac{P_{ANE}}{P_{RTN}}\right)$$

where $P_{ANE}$ belongs to the anomalous event in question and $P_{RTN}$ is the power of the surrounding RTN.

All the casuistry of the calculation is detailed in [52]. Finally, it is worth mentioning that the SNR of a particular ANE could be negative if the power of the surrounding RTN is higher than the power of the ANE itself. This may happen in cases where RTN masks other low-energy sounds, e.g., birds, because of the fluctuation of the road pass-bys.

#### 3.3.2. Impact Calculation

The computation of the ANE impact consists of determining the contribution of a particular event to the equivalent noise level of the recorded audio after applying the A-weighting filter [53], i.e., $L_{Aeq}$. This metric has been defined to evaluate the effect of each ANE on the noise map $L_{Aeq}$. It is calculated as the difference between the $L_{Aeq}$ computed with all the raw data and the $L_{Aeq}$ after removing the ANE by means of a linear interpolation (see Equation (3)). The event should be replaced by a lower-period linear interpolation to maintain the weight of the surrounding RTN level. That way, the road-traffic noise measurement is as accurate as possible in the whole integration time.

(3) $$\Delta L_{Aeq}=L_{Aeq,ANE}-L_{Aeq,\bar{ANE}}$$

where $\Delta L_{Aeq}$ is the contribution of this ANE to the $L_{Aeq}$, $L_{Aeq,ANE}$ is the A-weighted equivalent noise level of the raw audio given an evaluation period and $L_{Aeq,\bar{ANE}}$ is the equivalent A-weighted equivalent noise level of the same audio after removing the ANE.

In the DYNAMAP project, noise-map values are updated every 5 min, thus the contribution of ANEs on the $L_{Aeq}$ will be evaluated in this integration time, i.e., $\Delta L_{A300s}$. The low-level interpolations are carried out in a 1-s integration window, i.e., $L_{A1s}$, as it heuristically proved to be a good trade-off between representing all audible short events and not adding imperceptible changes to the equivalent sound measurement. As the goal is to obtain the equivalent noise level completely unaffected by the ANE, a span of 500 ms is left before and after the exact ANE label. More details on the casuistry of this calculation may be found in [52].

Finally, it is worth mentioning that the $L_{A300s}$ measurement is conducted in a 5-min sliding window where the ANE is centered. This is a better approximation than using a 5-min fixed window as the mean distance between RTN samples and ANE samples is reduced. The fact that future samples are needed (plus the fact that the exact labeling can only be provided after listening and labeling the recordings) implies that this measurement can only be applied in an off-line analysis and not in the real-time operation mode of the WASN.

## 4. Dataset Analysis

In this section, a detailed analysis of the ANEs present in the labeled WASN-based audio database is described. Specifically, the distribution of the occurrences and duration of the ANE subcategories together with their contextual SNR distribution and the impact of each of them to the $L_{Aeq}$ value are analyzed. First, an analysis was conducted regarding the general characteristics of the new audio dataset considering the spectral and time behavior of nodes recordings along one day. Secondly, two more detailed analyses were performed: *(i)* aggregating values of occurrence, duration, and SNR, as well as $L_{Aeq}$ impact considering all network sensors at a whole and, *(ii)* highlighting the particularities of sensor’s locations of the WASN in terms of the same ANE statistical measures.

### 4.1. General Characteristics

The global trend of the audio database obtained from the sensor network has been firstly analyzed through focusing mainly in RTN, disregarding specific characteristics of the ANE observed during recordings. In this regard, spectrum-time profiles [54] defined as the hourly time evolution of the mean spectrum of the audio has been computed for each sensor and day, following the same approach as in [24]. Their computation has been performed using the 48 frequency sub-bands of the Mel-Frequency Cepstral Coefficients (MFCC) features of the incoming audio signal, following the setup explained in [55], and computing the mean spectrum along the 20 min of audio gathered every hour. This mean spectrum obtained for each of the 24 h in a day conforms the spectrum-time profile of a complete measured day, which mainly includes RTN raw signal.

**Figure 5:**
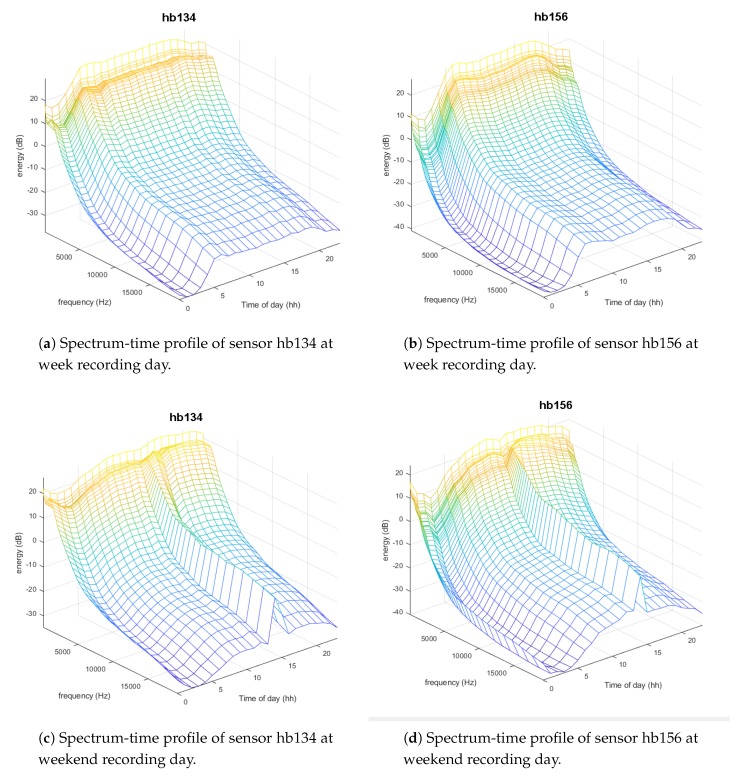
Spectrum-time profiles of sensors hb134 and hb156 at weekday (the 2nd of November 2017) and at weekend day (the 5th of November 2017).

Figure 5 shows four examples of the measured spectrum-time profiles, for two sensors hb134 and hb156 and for the two recorded days of the campaign. As can be observed, the general trend of the spectrum-time profile during weekday (Figure 5a,b) is quite similar for these two sensors, while sensor hb156 presents a slightly higher energy at high frequencies and during a narrower daily period. During the weekend (Figure 5c,d) a very persistent ANE of hard rain was present in almost all sensors, which can be seen as a high spectrum-profile value around 14 h. Additionally, another aspect that can be highlighted comparing week spectrum-time profiles with those of the weekend is that during weekend the raising and decreasing of acoustic energy during daily period is smoother than during the week day, which can be explained by the fact that on work days there are traffic rush hours at the beginning and ending of working hours.

### 4.2. Overall Analysis

Table 1 lists the distribution of the ANEs in terms of their number of occurrences observed within the recorded database, showing both the aggregated and segregated distributions for all sensors, respectively. The total amount of ANEs labeled in the dataset generation represents a 1.8% of all the recorded time, together with around 3.4% of raw data labeled as *cmplx*, which is the data result of the mixture of noises or with unidentifiable sound.

Table 1 shows the distribution of total ANE duration segregated by ANE subcategory. Most frequently observed ANEs were rain (6413.5 s) and thunder (753.4 s), due do the thunderstorm episode observed during the weekend day in the city of Rome at 14:00. Non-meteorological ANEs with higher total duration values were sounds of vehicles brakes (1387.2 s), trains (655.7 s), birds (338.9 s), sirens (327.8 s), horns (165.7 s), and sounds of trucks (382.4 s). ANEs with mid-total duration values were alarm sounds (84.3 s), interfering sounds (69.4 s), structure movement sounds (45.8 s), noise of pressurized air or *busd* (56.6 s), and music (31.6 s), while ANEs with lowest presence during the recordings were door sounds (16.3 s), airplanes (14.5 s), and bikes (9.6 s).

**Figure 6:**
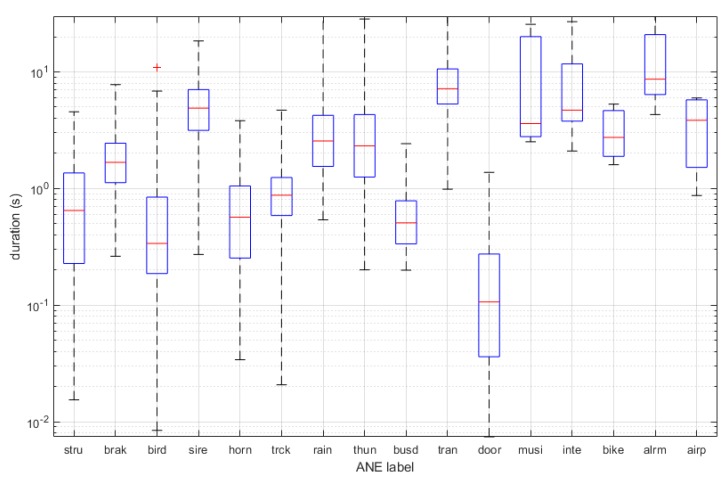
Boxplots of duration for each ANE category. Logarithmic axis of duration is used for a better observation of the duration values.

In Figure 6, the boxplots of the ANE durations are shown. The longer ANE correspond to *musi* and *alrm*, while the shorter are the *door*, *bird* and *busd*.

**Figure 7:**
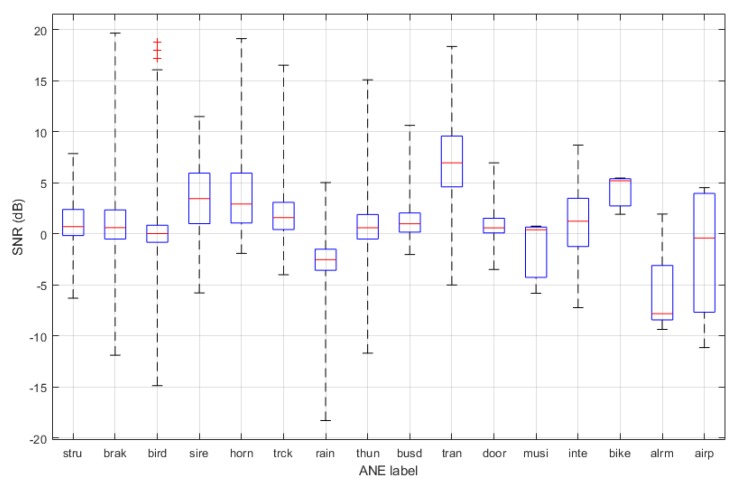
Boxplots of SNR for each ANE subcategory.

To determine the salience of the ANEs, the SNR measure is calculated by following the steps in Section 3.3.1. Figure 7 shows the SNR distributions for each ANE category. It can be observed that ANEs with higher SNR are sounds of trains, bikes, sirens, trucks, and horns.

### 4.3. ANEs’ Impact on the $L_{Aeq}$

The ANE contribution in the noise map can be analyzed in several ways. In this study, each ANE has been characterized with the two variables analyzed in the previous section: duration and SNR. Nevertheless, the impact of each ANE to the noise map should be quantified, and preliminary studies with a smaller dataset show that has a strong dependence on the SNR and the duration values [52]. The impact consists of the $L_{A300s}$ measurement of the raw audio minus the same measurement after removing the noise event, which allows discovery of the final contribution of the event to the noise map (more details are given in Section 3.3.2).

**Figure 8:**
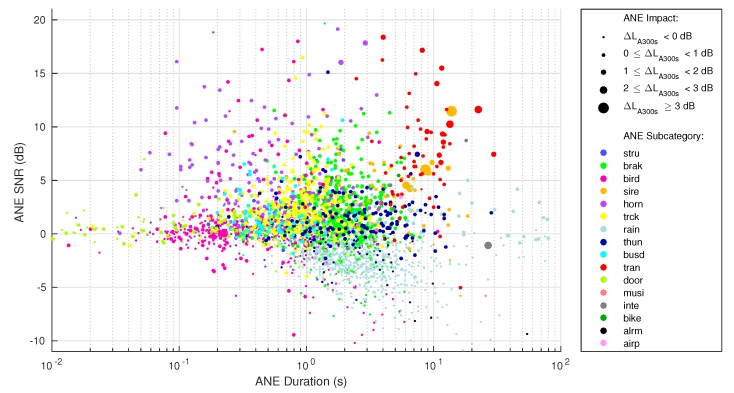
Scatterplot of all ANE parameters separated in subcategories (recorded and labeled in the 2nd and the 5th of November 2017).

To find a first approximation to the ANE subcategories and impact analysis, all the recorded ANEs are depicted according to their characteristics in Figure 8. The SNR is plotted on the vertical axis and the duration on the horizontal axis in a logarithmic scale, for illustration purposes. Also, the size of the marker represents the ANE impact, in a scale indicated in the legend. Besides, the class of the event is depicted in a color scale detailed in the legend, designed to distinguish events with similar parameters easily (the reader is referred to Section 3.2 for a list of all ANE subcategories).

In Figure 8 the reader may appreciate that the class distribution of the events is not uniform, as seen in Table 1. Events shorter than 1 s does not appear to have a significant impact on the $L_{A300s}$ when evaluated individually, as they represent an impact near 0 dB. However, events that last more than 4 s and have a positive SNR may contribute to the $L_{A300s}$ level with impacts of more than 3 dB. Among these significant ANEs, train pass-bys and sirens are the events presenting a higher overall impact score and presence, as also depicted in Figure 7 (where bike noise also appears to have a high median impact because it has only three occurrences). The events presenting a higher impact on the $L_{A300s}$ are trains and sirens, mainly, being only the trains the ANE that surpass the 3 dB level.

It is worth mentioning that in some long events, the SNR and the impact on the $L_{A300s}$ have computation problems. Hence, in Figure 8, only ANEs with both a quantifiable SNR and impact are depicted. Of the total of 2014 events, only three have a duration of more than 300 s, hence, making the impact on the $L_{A300s}$ impossible to calculate. In addition, 61 events give SNR calculation problems, mainly due to high ANE density segments where the interpolation between RTN labels is not possible. All those events have been discarded from these representations.

### 4.4. Node-Based Analysis

The main upgrade of the dataset detailed in this work, apart from the time distribution of the recordings and the total amount of time labeled, is the fact that the data gathered corresponds to 19 different locations and nodes in a WASN. This leads us to detail a spatial study of the collected data, assuming that not all nodes will observe the same subcategories of ANEs and, of course, the same number of occurrences. This is a key study for the final usage of this dataset, which is the training of the ANED, to detect the ANE in all the sensors of the WASN. A first approach to the homogeneity of this network will be given by the results of the cross analysis between ANE subcategory and sensor Id.

**Figure 9:**
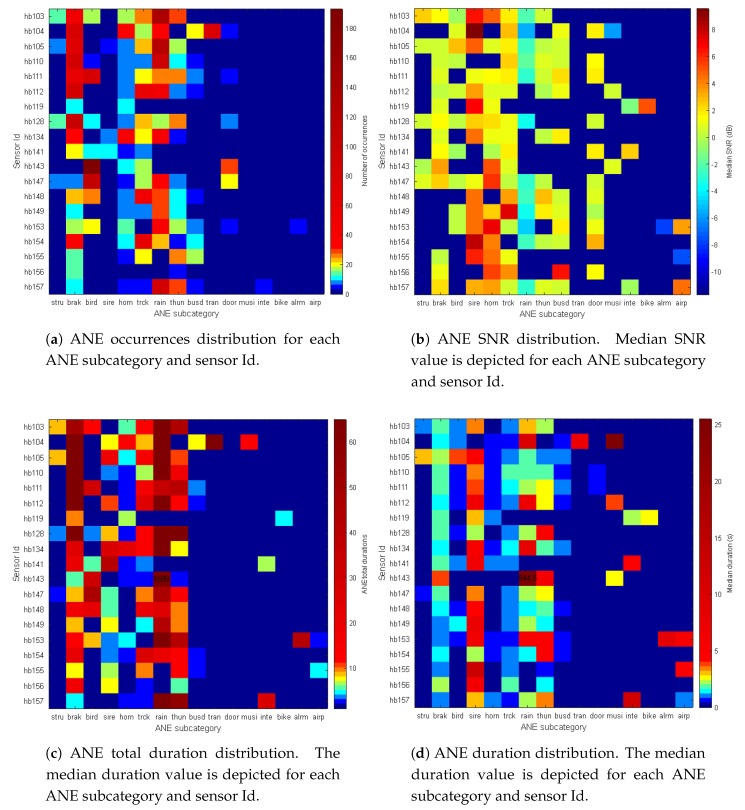
ANE parameters distribution per subcategory for each WASN node.

Figure 9a shows the ANE occurrences distribution segregated by sensors Id and ANE subcategory as an image, where it can be appreciated that birds in sensor hb143 are the ones more frequently observed, as birds produce short noise bursts and they can be very repetitive in certain locations and hours. Otherwise, the rain episode during the weekend day of the recording campaign is the second mostly observed event. From the same figure, it can be observed that noise events with quite uniform distribution across all the sensors network are some which are more related to traffic (horns, brakes, and sounds of trucks), and meteorological sounds during the weekend (rain and thunder). The rest of ANEs show a more irregular distribution across the sensor network during the recording campaign.

Figure 9b shows the median SNR values segregated by sensors and ANE subcategory. There are several nodes and ANE subcategories that exhibit positive SNR values, and their maximum values are attained for sirens, horns, trucks, and *buses* while medium SNRs are observed for brakes, birds, thunder, doors, and airplanes. The negative median SNR values of *rain* can be basically explained by the fact that the used computation methodology of SNR (see [23] for further details) is imprecise when the ANE duration is too high because of the underlying stationary assumption of the RTN assumed within this method.

Figure 9c shows the total duration of ANE recorded for each ANE type and sensor Id. The colormap scale leaves the maximum value as an outlier, depicted as a numeric value 1689 for rain in sensor hb143. Regarding the other values, it can be appreciated that *brak*, *sire*, *horn*, *trck*, *rain* and *thun* are the ANEs with more regular presence across the entire sensor network, while other ANEs like *stru*, *tran*, *musi*, *inte*, *bike*, and *alrm* are more irregularly observed. Figure 9d shows the ANE mean duration distribution per category for each of the WASN sensors, i.e., the median ANE duration statistic has been computed for each cell of the depicted matrix. The color bar legend also presents an outlier, which is reached by sensor hb143 and rain ANE category. The following maximum mean duration is related to sensor hb104 and *musi*.

As a conclusion of this WASN-based analysis, *siren* is the subcategory of ANEs with longest duration and with presence in most of the sensors, and so are *horns*, but the latter have shorter duration; both ANEs present high values of SNR. Another two subcategories are present in most of the sensors in this WASN-based recording campaign; *rain* and *thunder* have a wide presence in the recording, due to the fact that on the 5th of November nearly all the WASN suffered heavy rain in the afternoon. The main difference between them is that while *rain* presents mainly low SNR values, *thunder* presents mid SNR values. *birds* present values of high occurrence and duration for several sensors, but the SNR associated with the birdsong is moderate or low. Nevertheless, the main output of this analysis is that there are few events with uniform appearance in all the sensors, and that most of the subcategories labeled in this work correspond to recordings of fewer groups of sensors. This leads us to the conclusion that the WASN-based recording considering all the sensors in the network was a requirement to observe the variety of the distribution of the events occurring in the entire network.

## 5. Discussion and Conclusions

In this section, several key aspects of this work are discussed and concluded after the recording, labeling, and analysis of the WASN-based suburban scenario audio samples.

### 5.1. WASN-Based Dataset Analysis vs. Expert-Based Dataset

As previously stated, a preliminary suburban environment dataset in Rome was published in [23], which consisted of a set of expert-based recordings of limited duration and scope. The work presented in this article is based on the knowledge acquired from that first dataset. It responds to the need for increasing the coverage of the RTN and the ANEs at all hours of the day and night, the weekend, and even when elements external to the noise appear in the measurements, such as adverse weather conditions, i.e., in real-operation conditions.

The WASN-based dataset presented in this work has been enriched with seven classes in comparison to the previous one: *rain*, *thun*, *tran*, *bird*, *alrm*, *inte* and *airp*. On the contrary, it has not been possible to record the noise of people talking (*peop*) as in the previous dataset caused by the presence of workers in the portals. All the rest of the ANEs were already part of the expert-based dataset, but with fewer occurrences because the dataset was much smaller. The preliminary dataset contained 3.2% of ANE of the total recorded time, and this new dataset contains 1.8%. A possible explanation to these differences is that the expert-based dataset recording was centered in daytime and this WASN-based dataset has recorded day and night, where night shows low presence of ANE with respect to the day.

The longest ANEs have been found within the *sire* subcategory, while the shortest ones are found in the *door* subcategory, as also happened in the expert-based recording. Moreover, the ANEs labeled as *horn* and *sire* present the highest SNR in both datasets. However, it is worth noting that in this new dataset there are samples of *tran* and *inte* subcategories that also entail high SNRs in many occurrences, a characteristic that was not found in [23].

From this comparative analysis, it can be deduced that the data captured in the expert-based dataset was suitable enough for the first characterization of the suburban soundscape. Nevertheless, the WASN-based recording campaign has shown that there were several noise subcategories that in the preliminary recording campaign had not been recorded and labeled, which present critical characteristics in terms of SNR and duration.

### 5.2. WASN-Based Dataset and Node Homogeneity

The final use of the presented dataset is the training of the ANED algorithm for a precise detection of the ANEs in all the nodes of the WASN already deployed in the Rome pilot area. Although the developed dataset contains a significantly larger sample of both RTN and ANEs—around 19 times the data gathered in the preliminary expert-based dataset [23]—the analysis of the acoustic data confirms the heterogeneous distribution of the ANEs subcategories in real-life environments already observed in [23]. This heterogeneity has also been observed across the nodes as detailed in Section 4.4. Although some of the subcategories occurred in most of the nodes (e.g., *brak*, *rain*, *truck* and *horn*), there are others found particular in some sensors of the network (e.g., *airp*, *bike* and *train*).

The design of a WASN-based dataset raises the hypothesis of homogeneity of the raw data captured in each of the nodes of the network. This hypothesis was analyzed by means of the distribution of the ANEs in the previous recording campaign [56], with an analysis of the five recording locations. To ensure that the ANED will operate properly in all the nodes, their acoustic environments should present a certain homogeneity in terms of frequency distribution. To collect the data in similar conditions, all nodes have been installed maintaining the same distances and orientations to the road. Nevertheless, a study to evaluate the homogeneity should be carried out taking into account both the locations of the nodes and the occurrences of the ANEs with the final goal of a generalist training of ANED for all the network.

### 5.3. Impact on the $L_{A300s}$ of the ANE Subcategories

In this paper, the impact on the computation of $L_{A300s}$ has been evaluated for every individual ANE every 5 min. The analysis presents interesting results to be discussed considering the SNR and duration of individual ANEs. The results in Section 4 show the existence of several ANEs with high impact, which present high SNR and long duration. After the individual analysis of the impact of ANEs on the $L_{A300s}$ computation, future work will also take into account the fact that dynamic acoustic mapping in real-life conditions face a more complex operating scenario. In a real-operation scenario, several ANEs can occur in a predefined integration time, so the ANE impact must be evaluated in an aggregated way for each period.

Another relevant result of the individual ANE analysis is the presence of ANEs with negative SNR. As detailed in Section 3.3.1, the SNR is evaluated taking into account each ANE in relation to its surrounding RTN signal level. In certain cases, the RTN decreases as the ANE occurs, so a negative SNR is obtained. Therefore, only those events with positive SNR should be removed from the $L_{Aeq}$ computation, as also concluded in [52].

From this work, it can be concluded that working with data recorded in a real operating scenario is crucial to obtain a reliable modelling of the nodes’ acoustic environment, according to the differences observed between the expert- and WASN-based datasets. Moreover, the analysis of the WASN-based collected data again shows the important role played by SNR and duration of individual ANEs in their impact on the $L_{Aeq}$ computation, obtaining ANEs that should be considered for their high impact on the equivalent level.

## Figures and Tables

**Table 1:** Aggregated ANE occurrences and their total duration distribution per category.

Category	Number of Occurrences	Total Duration (s)
ANE	3170	10,752.9
rain	754	6413.5
brak	737	1387.2
thun	236	753.4
tran	76	655.7
trck	380	382.4
bird	482	338.9
sire	55	327.8
horn	210	165.7
alrm	5	84.3
inte	8	69.4
busd	88	56.6
stru	46	45.8
musi	3	31.6
door	83	16.3
airp	4	14.5
bike	3	9.6

## Abbreviations

The following abbreviations are used in this manuscript:

AED	Audio Event Detector
ANE	Anomalous Noise Event
ANED	Anomalous Noise Event Detection
BCK	Background Noise
CASA	Computational Scene and Event Analysis
CLEAR	Classification of Events, Activities and Relationships
DNN	Deep Neural Networks
DYNAMAP	Dynamic Noise Mapping
END	European Noise Directive
GTCC	Gammatone Cepstral Coefficients
MFCC	Mel-Frequency Cepstral Coefficients
RTN	Road-Traffic Noise
SED	Sound Event Detector
SONYC	Sounds of New York City
SNR	Signal-to-Noise Ratio
VPN	Virtual Private Network
WASN	Wireless Acoustic Sensor Network

## References

[1] Goines L., Hagler L. (2007). Noise Pollution: A Modern Plague. South. Med. J. Birm. Ala..

[2] Guite H., Clark C., Ackrill G. (2006). The impact of the physical and urban environment on mental well-being. Public Health.

[3] Hänninen O., Knol A.B., Jantunen M., Lim T.A., Conrad A., Rappolder M., Carrer P., Fanetti A.C., Kim R., Buekers J. (2014). Environmental burden of disease in Europe: Assessing nine risk factors in six countries. Environ. Health Perspect..

[4] Bello J.P., Silva C. (2019). SONYC: A System for Monitoring, Analyzing, and Mitigating Urban Noise Pollution. Commun. ACM.

[5] EU (2002). Directive 2002/49/EC of the European Parliament and the Council of 25 June 2002 relating to the assessment and management of environmental noise. Off. J. Eur. Commun..

[6] Manvell D. (2015). Utilising the Strengths of Different Sound Sensor Networks in Smart City Noise Management. Proceedings of the EuroNoise 2015.

[7] Alías F., Alsina-Pagès R.M. (2019). Review of Wireless Acoustic Sensor Networks for Environmental Noise Monitoring in Smart Cities. J. Sens..

[8] Nencini L., De Rosa P., Ascari E., Vinci B., Alexeeva N. SENSEable Pisa: A wireless sensor network for real-time noise mapping. Proceedings of the EuroNoise 2012.

[9] Botteldooren D., De Coensel B., Oldoni D., Van Renterghem T., Dauwe S., Mee D., Hillock I.D. (2011). Sound monitoring networks new style. Proceedings of the Acoustics 2011.

[10] Mietlicki F., Mietlicki C., Sineau M. (2015). An innovative approach for long-term environmental noise measurement: RUMEUR network. Proceedings of the EuroNoise 2015.

[11] Camps-Farrés J., Casado-Novas J. (2018). Issues and challenges to improve the Barcelona Noise Monitoring Network. Proceedings of the EuroNoise 2018.

[12] Bain M. (2014). SENTILO—Sensor and Actuator Platform for Smart Cities. https://joinup.ec.europa.eu/document/sentilo-sensor-and-actuator-platform-smart-cities.

[13] Sevillano X., Socoró J.C., Alías F., Bellucci P., Peruzzi L., Radaelli S., Coppi P., Nencini L., Cerniglia A., Bisceglie A. (2016). DYNAMAP—Development of low cost sensors networks for real time noise mapping. Noise Mapp..

[14] De la Piedra A., Benitez-Capistros F., Dominguez F., Touhafi A. Wireless sensor networks for environmental research: A survey on limitations and challenges. Proceedings of the 2013 IEEE EUROCON.

[15] Rawat P., Singh K.D., Chaouchi H., Bonnin J.M. (2014). Wireless Sensor Networks: A Survey on Recent Developments and Potential Synergies. J. Supercomput..

[16] Bertrand A. Applications and trends in wireless acoustic sensor networks: A signal processing perspective. Proceedings of the 18th IEEE Symposium on Communications and Vehicular Technology in the Benelux (SCVT).

[17] Griffin A., Alexandridis A., Pavlidiand D., Mastorakis Y., Mouchtaris A. (2015). Localizing multiple audio sources in a wireless acoustic sensor network. Signal Process..

[18] Socoró J.C., Alías F., Alsina-Pagès R.M. (2017). An Anomalous Noise Events Detector for Dynamic Road Traffic Noise Mapping in Real-Life Urban and Suburban Environments. Sensors.

[19] Wang D., Brown G.J. (2006). Computational Auditory Scene Analysis: Principles, Algorithms, and Applications.

[20] Giannoulis D., Benetos E., Stowell D., Rossignol M., Lagrange M., Plumbley M.D. Detection and classification of acoustic scenes and events: An IEEE AASP challenge. Proceedings of the 2013 IEEE Workshop on Applications of Signal Processing to Audio and Acoustics.

[21] Foggia P., Petkov N., Saggese A., Strisciuglio N., Vento M. (2015). Reliable detection of audio events in highly noisy environments. Pattern Recognit. Lett..

[22] Mesaros A., Diment A., Elizalde B., Heittola T., Vincent E., Raj B., Virtanen T. (2019). Sound event detection in the DCASE 2017 Challenge. IEEE/ACM Trans. Audio Speech Language Process..

[23] Alías F., Socoró J.C. (2017). Description of anomalous noise events for reliable dynamic traffic noise mapping in real-life urban and suburban soundscapes. Appl. Sci..

[24] Socoró J.C., Alsina-Pagès R.M., Alías F., Orga F. Adapting an Anomalous Noise Events Detector for Real-Life Operation in the Rome Suburban Pilot Area of the DYNAMAP’s Project. Proceedings of the EuroNoise.

[25] Nakajima Y., Sunohara M., Naito T., Sunago N., Ohshima T., Ono N. (2016). DNN-based Environmental Sound Recognition with Real-recorded and Artificially-mixed Training Data. Proceedings of the 45th International Congress and Exposition on Noise Control Engineering (InterNoise 2016).

[26] Mesaros A., Heittola T., Virtanen T. TUT database for acoustic scene classification and sound event detection. Proceedings of the 24th European Signal Processing Conference (EUSIPCO 2016).

[27] Stowell D., Giannoulis D., Benetos E., Lagrange M., Plumbley M.D. (2015). Detection and Classification of Acoustic Scenes and Events. IEEE Trans. Multimed..

[28] Heittola T., Mesaros A., Eronen A., Virtanen T. (2013). Context-dependent sound event detection. EURASIP J. Audio Speech Music Process..

[29] Foggia P., Petkov N., Saggese A., Strisciuglio N., Vento M. (2016). Audio Surveillance of Roads: A System for Detecting Anomalous Sounds. IEEE Trans. Intell. Transp. Syst..

[30] Zambon G., Benocci R., Bisceglie A., Roman H.E., Bellucci P. (2017). The LIFE DYNAMAP project: Towards a procedure for dynamic noise mapping in urban areas. Appl. Acoust..

[31] Bellucci P., Peruzzi L., Zambon G. (2017). LIFE DYNAMAP project: The case study of Rome. Appl. Acoust..

[32] Stowell D., Plumbley M.D. (2013). An open dataset for research on audio field recording archives: Freefield1010. arXiv.

[33] Salamon J., Jacoby C., Bello J.P. A dataset and taxonomy for urban sound research. Proceedings of the 22nd ACM International Conference on Multimedia.

[34] Socoró J.C., Ribera G., Sevillano X., Alías F. (2015). Development of an Anomalous Noise Event Detection Algorithm for dynamic road traffic noise mapping. Proceedings of the 22nd International Congress on Sound and Vibration (ICSV22).

[35] Piczak K.J. ESC: Dataset for Environmental Sound Classification. Proceedings of the 23rd ACM International Conference on Multimedia.

[36] Rossignol M., Lafay G., Lagrange M., Misdariis N. SimScene: A web-based acoustic scenes simulator. Proceedings of the 1st Web Audio Conference (WAC).

[37] Gloaguen J.R., Can A., Lagrange M., Petiot J.F. Estimating Traffic Noise Levels using Acoustic Monitoring: A Preliminary Study. Proceedings of the Detection and Classification of Acoustic Scenes and Events 2016 (DCASE’2016).

[38] Çakır E., Parascandolo G., Heittola T., Huttunen H., Virtanen T. (2017). Convolutional Recurrent Neural Networks for Polyphonic Sound Event Detection. IEEE/ACM Trans. Audio Speech Lang. Process..

[39] Salamon J., MacConnell D., Cartwright M., Li P., Bello J.P. Scaper: A library for soundscape synthesis and augmentation. Proceedings of the 2017 IEEE Workshop on Applications of Signal Processing to Audio and Acoustics (WASPAA).

[40] Salamon J., Bello J.P. (2017). Deep Convolutional Neural Networks and Data Augmentation for Environmental Sound Classification. IEEE Signal Process. Lett..

[41] Temko A., Malkin R., Zieger C., Macho D., Nadeu C., Omologo M., Stiefelhagen R., Garofolo J. (2007). CLEAR Evaluation of Acoustic Event Detection and Classification Systems. Multimodal Technologies for Perception of Humans: First International Evaluation Workshop on Classification of Events, Activities and Relationships, CLEAR 2006.

[42] Temko A. (2007). Acoustic Event Detection and Classification. Ph.D. Thesis.

[43] Heittola T., Klapuri A., Stiefelhagen R., Bowers R., Fiscus J. (2008). TUT Acoustic Event Detection System 2007. Multimodal Technologies for Perception of Humans. International Evaluation Workshops CLEAR 2007 and RT 2007.

[44] Van Grootel M.W.W., Andringa T.C., Krijnders J.D. DARES-G1: Database of Annotated Real-world Everyday Sounds. Proceedings of the NAG/DAGA Meeting 2009.

[45] Giannoulis D., Stowell D., Benetos E., Rossignol M., Lagrange M., Plumbley M.D. A database and challenge for acoustic scene classification and event detection. Proceedings of the 21st European Signal Processing Conference (EUSIPCO 2013).

[46] Mesaros A., Heittola T.K., Benetos E., Foster P., Lagrange M., Virtanen T., Plumbley M.D. (2018). Detection and Classification of Acoustic Scenes and Events: Outcome of the DCASE 2016 Challenge. IEEE/ACM Trans. Audio Speech Lang. Process..

[47] Mesaros A., Heittola T., Diment A., Elizalde B., Shah A., Vincent E., Raj B., Virtanen T. DCASE 2017 challenge setup: Tasks, datasets and baseline system. Proceedings of the DCASE 2017-Workshop on Detection and Classification of Acoustic Scenes and Events.

[48] Nencini L. (2015). DYNAMAP monitoring network hardware development. Proceedings of the 22nd International Congress on Sound and Vibration (ICSV22).

[49] Nencini L. Progetto e realizzazione del sistema di monitoraggio nell’ambito del progetto Dynamap. Proceedings of the 44∘ Congress of the Italian Acoustic Association.

[50] International Electroacoustics Commission (2013). Electroacoustics-Sound Level Meters-Part 1: Specifications (IEC 61672-1).

[51] Nencini L., Bisceglie A., Bellucci P., Peruzzi L. Identification of failure markers in noise measurement low cost devices. Proceedings of the 45th International Congress and Exposition on Noise Control Engineering (InterNoise 2016).

[52] Orga F., Alías F., Alsina-Pagès R.M. (2017). On the Impact of Anomalous Noise Events on Road Traffic Noise Mapping in Urban and Suburban Environments. Int. J. Environ. Res. Public Health.

[53] Pierre R.L.S., Maguire D.J. The impact of A-weighting sound pressure level measurements during the evaluation of noise exposure. Proceedings of the NOISE-CON 2004.

[54] Socoró J.C., Alsina-Pagès R.M., Alías F., Orga F. (2019). Acoustic Conditions Analysis of a Multi-Sensor Network for the Adaptation of the Anomalous Noise Event Detector. Proceedings.

[55] Valero X., Alías F. (2012). Gammatone Cepstral Coefficients: Biologically Inspired Features for Non-Speech Audio Classification. IEEE Trans. Multimed..

[56] Orga F., Alsina-Pagès R.M., Alías F., Socoró J.C., Bellucci P., Peruzzi L. Anomalous Noise Events Considerations for the Computation of Road Traffic Noise Levels in Suburban Areas: The DYNAMAP’s Rome Case Study. Proceedings of the 44° Congress of the Italian Acoustic Association.

